# Unsupervised blind image quality assessment via joint spatial and transform features

**DOI:** 10.1038/s41598-023-38099-5

**Published:** 2023-07-05

**Authors:** Chao Yang, Qinglin He, Ping An

**Affiliations:** School of Communication and Information Engineering, Shanghai University, Shanghai, China

**Keywords:** Engineering, Electrical and electronic engineering

## Abstract

A novel unsupervised blind image quality assessment (BIQA) method, which requires no mean opinion scores for model training is presented in this paper. The method employs joint spatial and transform features as quality degradation metrics, specifically, phase congruency, gradient magnitude (GM), and GM and Laplacian of Gaussian response and local normalized coefficient are extracted as spatial features, and Karhunen–Loéve transform coefficient and discrete cosine transform coefficient are modeled as transform features. Both spatial and transform features are well analyzed to remove the redundancy, and then fitted to the multivariate Gaussian model for no-reference image quality assessment. Extensive experiments conducted on seven IQA databases demonstrate the superiority of the proposed method over the state-of-the-art both supervised and unsupervised BIQA methods.

## Introduction

The quality of images plays a more important role with the development of multimedia and transmission technologies, efforts to accurately assess image quality with lower complexity have become increasingly important. As human is the ultimate receiver of images, subjective quality assessment always has the highest accuracy but is time-consuming and expensive, making it impractical for most applications. On the contrary, objective quality assessment attempts to assess image quality without human involvement, which is more practical for real-world use and has therefore become increasingly important.

Objective image quality assessment (IQA) can be classified into three categories based on the usage of reference information, namely full-reference (FR) IQA^1–4^, reduced-reference (RR) IQA^5–7^, and no-reference IQA (NR-IQA)/blind IQA (BIQA)^8–11^. FR-IQA requires complete access to the reference image and compares it with the distorted image using a distortion measure for quality assessment. RR-IQA utilizes a subset of pre-determined features from both the reference and distorted images for quality assessment and requires less reference information than FR-IQA but more than BIQA. BIQA predicts the perceived quality of an image by extracting and analyzing its internal features, requiring no reference. This makes BIQA highly practical in real-world applications where reference images may be limited or unavailable.

BIQA methods can be further classified into two categories based on the usage of training labels. Supervised BIQA methods usually use subjective scores as labels to train the quality assessment models, and the difference mainly exists in the features and regression models used. For example, Mittal et al.^8^ extracted mean subtracted contrast normalized (MSCN) feature, Yang et al.^12,13^ extracted and enhanced naturalness and structural features via Karhunen-Loéve transform (KLT), while Zhang et al.^14^ proposed to extract quality-aware features from joint generalized local binary pattern statistics. These features were then mapped to subjective scores via support vector regression (SVR) to perform BIQA. Min et al.^15^ proposed to utilize multiple pseudo reference images created through various types and levels of distortion aggravation with FR-IQA method to generate similarity scores for BIQA.

With the advent of deep learning technologies, several approaches have been proposed for BIQA that utilize convolutional neural networks (CNN) for end-to-end joint feature extraction and regression. Ma et al.^16^ and Zhu et al.^17^ employed CNNs for this purpose. Wang et al.^18^ proposed a dual-perception network (DPNet) that uses end-to-end multi-task learning with knowledge distillation, while Lan et al.^19^ developed a framework that combines two feature extraction networks and a multilevel feature fusion (MFF) network to obtain multilevel degradation features for BIQA. Additionally, Wang et al.^20^ reformulated BIQA as an ordinal regression problem and achieved improved prediction accuracy by using deep CNNs and Transformers. Madhusudana et al.^21^ performed the prediction of distortion type and degree as an auxiliary task to learn features for BIQA. Pan et al.^22^ introduced a distortion aware module in CNN to perform BIQA on different distortions. Chen et al.^23^ propose an NR-IQA method via feature level pseudo-reference hallucination. Pan et al.^24^ proposed a multi-branch convolutional neural network to perform NR-IQA. Zhou et al.^25^ employed self-attention and recurrent neural network (RNN) to perform BIQA. Liu et al.^26^ proposed spatial optimal-scale filtering analysis for deep learning-based BIQA. Cao et al.^27^ proposed objective audio-visual quality assessment using attentional neural networks. Yu et al.^28^ employed transformers with self-attention mechanisms to perform NR-IQA.

Compared with supervised BIQA methods, unsupervised ones require no subjective scores for model parameters training and usually have better generalization ability. Wu et al.^9^ extracted one dimension feature from image binary patterns and utilized this feature to perform unsupervised BIQA with extremely low complexity. Natural image quality evaluator (NIQE)^29^ and its extensions, e.g., ILNIQE^30^ and SNP-NIQE^31^ employed multivariate Gaussian (MVG) model to perform unsupervised BIQA with different features. Venkatanath et al.^32^ proposed to estimate quality from spatial regions that are perceptually significant for unsupervised BIQA. Wu et al.^33^ proposed to consider the correlation between different color channels through quaternion representation, and then adopt MVG model to perform unsupervised BIQA. Also, Wu et al.^34^ incorporated histogram and deep-learned features along with natural scene statistical features to assess distortions. With the development of quality-aware features, the performance of the unsupervised BIQA method can be further improved.

The main contributions of this paper are summarized as follows:

- We propose an unsupervised BIQA method, to evaluate image quality from multiple aspects, we extract phase congruency (PC), gradient magnitude (GM), GM and Laplacian of Gaussian response (GM-LOG) to measure image structure, local normalized coefficient to measure image naturalness. Additionally, we extract KLT and discrete cosine transform (DCT) coefficients to measure image perception quality from the transform domain.
- Since we utilized multiple features in our method to reflect the characteristics of the HVS, ablation studies are conducted to analyze the contributions of different features, and comprehensive analyses are performed to select the most significant features to remove the redundancy of these multiple features.

**Figure 1:**
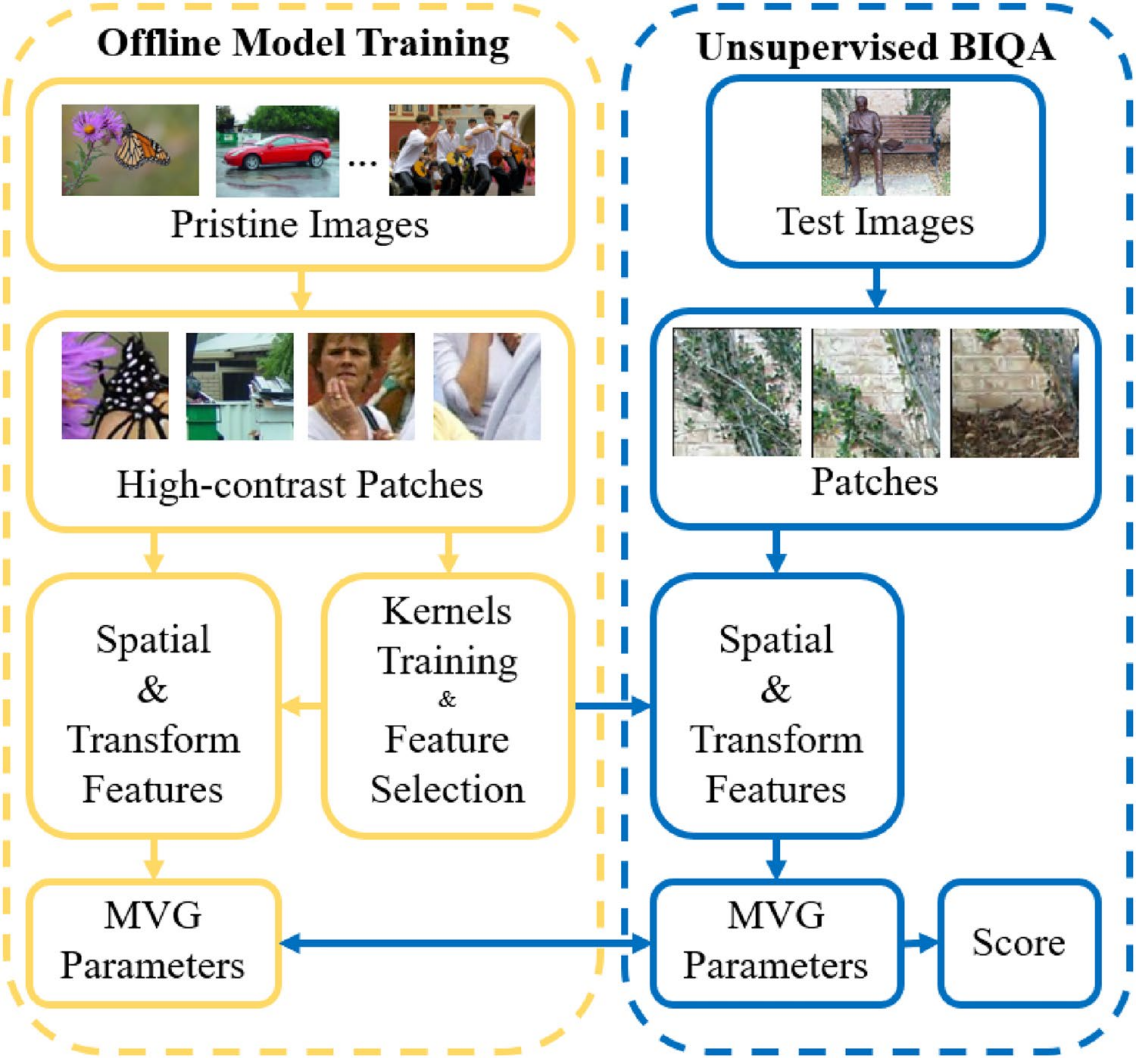
Framework of proposed method.

**Figure 2:**
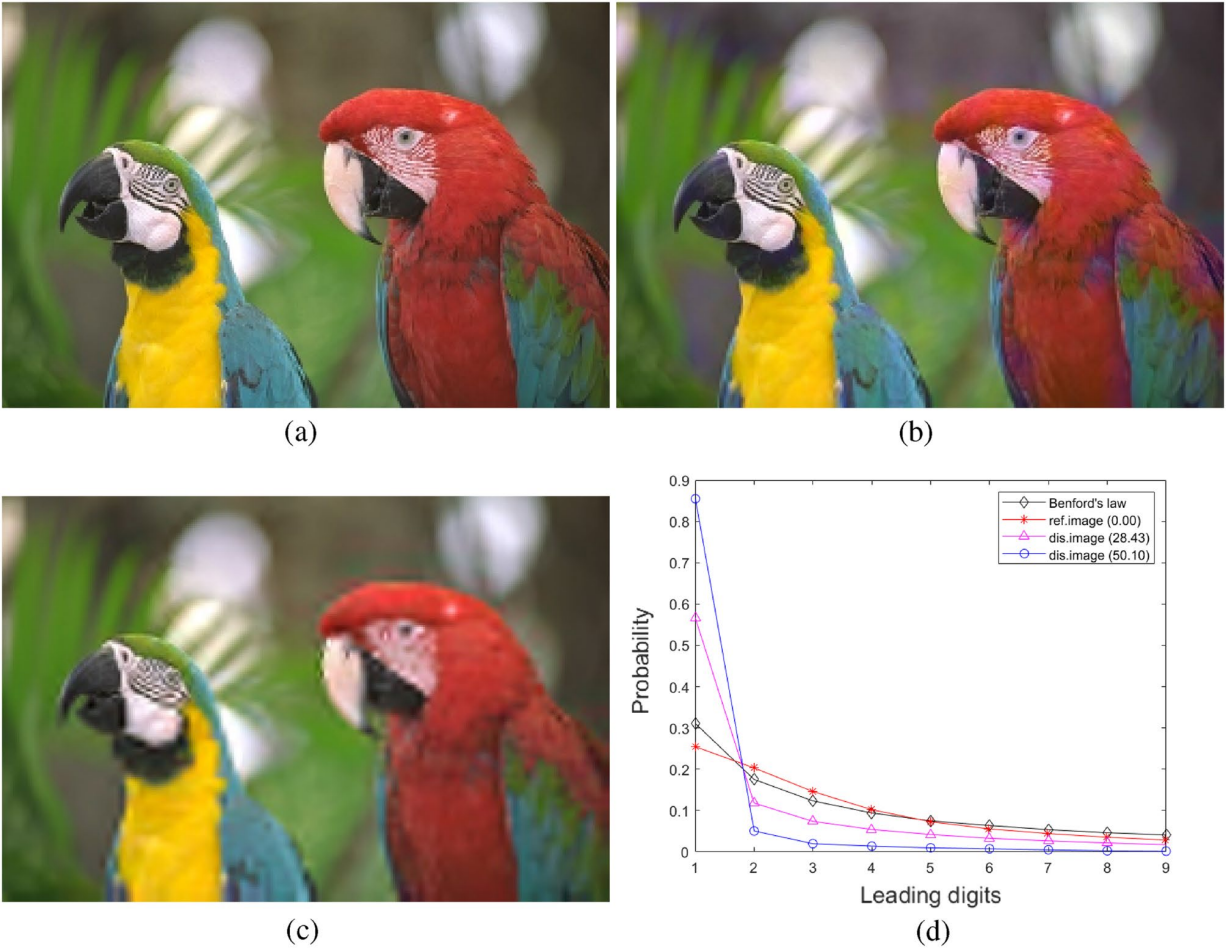
An example of Benford’s law in distorted images (**a**) reference image, (**b**) and (**c**) fast fading Rayleigh with DMOS being 28.43 and 50.10, (**d**) distribution of leading digits of DCT coefficients.

## Method

The framework of the proposed method is shown in Fig. 1. To better measure the perceived quality of an image, we extract spatial and transform features from non-overlapping image patches to form the feature matrix. Herein, to avoid the impact of non-texture patches, we only utilize high-contrast patches in pristine images for model training. During testing, we consider all patches from the test image. We then fit the MVG model with the mean vector $\nu$ and covariance matrix $\Sigma$ of the feature matrix.

We calculate the local and global qualities of distorted image as the distance between the pristine and distorted MVG model parameters:

1 $$\begin{aligned} q_{local}= & {} \frac{1}{n}\sum _{i=1}^{n}\sqrt{\left( {\nu _{p}}-{f_{i}}\right) ^{T}\left( \frac{\Sigma _{p}+\Sigma _{d}}{2}\right) ^{-1}\left( {\nu _{p}}-{f_{i}}\right) }~, \end{aligned}$$

2 $$\begin{aligned} q_{global}= & {} \sqrt{\left( {\nu _{p}}-{\nu _{d}}\right) ^{T}\left( \frac{\Sigma _{p}+\Sigma _{d}}{2}\right) ^{-1}\left( {\nu _{p}}-{\nu _{d}}\right) }~, \end{aligned}$$

where *n* is the number of image patches in each distorted image and $f_i$ is the corresponding feature vector, $\nu _{p}$, $\nu _{d}$ and $\Sigma _{p}$, $\Sigma _{d}$ are the mean vectors and covariance matrices of pristine image MVG model and distorted image MVG model, respectively.

Finally, the quality of the distorted image is measured as the weighted average of local and global quality as follows:

3 $$\begin{aligned} Q = \alpha * q_{global} + (1-\alpha ) * q_{local}~, \end{aligned}$$

where we set $\alpha$ = 0.5 in this paper based on experiments on seven IQA databases as demonstrated in “Parameters optimization and ablation studies”.

### Spatial features

An image is a two-dimensional (2D) spatial signal, and therefore, we extract spatial features from images to measure their perceptual quality. Specifically, since the perceived quality of an image is highly dependent on its structure and naturalness, we extract image structure-related features such as PC, GM, and GM-LOG features, along with naturalness-related features like MSCN.

PC is utilized as an indicator of the edge strength of an image, which is highly related to image structure. And we adopt the method in^35^ to compute the PC value of image *I* at position *p* as follows:

4 $$\begin{aligned} PC(p)=\frac{\sum _{f}H_{\theta _{f}}(p)}{\varepsilon +\sum _{f} \sum _{n} A_{n,\theta _{f}}(p)}~, \end{aligned}$$

where $H_{\theta _{f}}(p)=\sqrt{E_{n,\theta _{f}}(p)^{2}+O_{n,\theta _{f}}(p)^{2}}$, $E_{n,\theta _{f}}(p)=\sum _{n} e_{n,\theta _{f}}(p)$, $O_{n,\theta _{f}}(p)=\sum _{n} o_{n,\theta _{f}}(p)$, $A_{n,\theta _{f}}(p)=\sqrt{e_{n,\theta _{f}}(p)^{2}+o_{n,\theta _{f}}(p)^{2}}$, $e_{n,\theta _{f}}(p)$ and $o_{n,\theta _{f}}(p)$ are responses of even and odd-symmetric filters, *n* and $\theta _{f}$ are scale and directions, and $\varepsilon$ is a small positive constant. The color relevant space *O*^36^ is utilized to extract *PC*,

5 $$\begin{aligned} \left[ \begin{array}{l} O_{1} \\ O_{2} \\ O_{3} \end{array}\right] =\left[ \begin{array}{ccc} 0.06 &{} 0.63 &{} 0.27 \\ 0.30 &{} 0.04 &{} -0.35 \\ 0.34 &{} -0.6 &{} 0.17 \end{array}\right] \left[ \begin{array}{l} R \\ G \\ B \end{array}\right] ~, \end{aligned}$$

and the coefficients are fitted with Weibull distribution to form the feature vector.

GM represents the contrast of an image, which can highly affect the perceived quality of the image, therefore is an indispensable IQA index. We utilize filters $\varvec{D}_{h}=[1,-1]$ and $\varvec{D}_{v}=\varvec{D}_{h}^{T}$ to extract GM:

6 $$\begin{aligned} GM = \sqrt{(I * \varvec{D}_{h})^2 + (I * \varvec{D}_{v})^2}~, \end{aligned}$$

where $*$ is the convolution operation, and we compute the GM in the luminance channel. The distribution of GM coefficient is modeled with the Weibull distribution to form a feature vector.

Besides, we also extract finely selected GM-LOG features as structure features. We utilize Eq. (6) to extract the GM feature, with $D_{h}$ and $D_{v}$ being the Gaussian partial derivative filters in the horizontal and vertical directions, respectively. LOG feature is calculated as follows:

7 $$\begin{aligned} L = I * h_{LOG}~, \end{aligned}$$

where $h_{LOG}$ is the two-dimensional Laplacian of Gaussian operator. Then, the normalized *GM* and *L* are quantized into 10 levels, and the normalized bivariate histogram is calculated get the GM-LOG feature vector. We extract the GM-LOG feature from pristine images in^29^ and select the indices with the values all higher than 0.05 to remove the redundancy.

Image naturalness generally refers to the degree to which an image appears to be natural or realistic, and is a factor that can affect the perceived quality of an image. Therefore, it is often considered in IQA. The MSCN coefficient distribution of distorted natural scene images differs from pristine ones. Therefore, we employ it to measure the naturalness of an image, where MSCN is computed as:

8 $$\begin{aligned} M(i,j)=\frac{I(i,j)-\mu (i,j)}{\sigma (i,j)+1}~, \end{aligned}$$

where *i* and *j* are the pixel coordinates, $\mu$ and $\sigma$ are calculated as follows:

9 $$\begin{aligned} \mu (i,j)= & {} \sum _{l=-3}^{3}\sum _{k=-3}^{3}\omega _{l,k}I(i+l,j+k)~, \end{aligned}$$

10 $$\begin{aligned} \sigma ^{2}(i,j)= & {} \sum _{l=-3}^{3}\sum _{k=-3}^{3}\omega _{l,k}[I(i+l,j+k)-\mu (i,j)]^{2}~, \end{aligned}$$

and $\omega$ is a unit-volume Gaussian window with size 7 $\times$ 7. General Gaussian distribution (GGD) in^37^ is utilized to model the MSCN coefficient distribution as features. Besides, asymmetric generalized Gaussian distribution (AGGD) is employed to model the adjacent MSCN coefficient in four directions, including horizontal, vertical, main diagonal, and sub-diagonal^8^ as feature representation.

**Table 1:** SROCC results on different IQA databases, the best results of both supervised and unsupervised methods are boldfaced.

SROCC	LIVE	MICT	CSIQ_sub	TID2013_sub	KADID-10k_sub	CID2013	LIVE-C	W. A.
BRISQUE	–	0.8649 (− 1)	0.8992 (− 1)	0.8401 (− 1)	0.8163 (1)	0.4409 (1)	0.3137 (1)	0.6626
CORNIA	–	0.8640 (0)	0.8943 (0)	0.8604 (0)	0.8931 (0)	0.5850 (1)	**0.5365**(− 1)	**0.7642**
MEON	–	0.9300 (0)	**0.9300**(0)	**0.9012**(1)	**0.9171**(− 1)	0.3813 (0)	0.3640 (1)	0.7186
RankIQA	–	**0.9109**(0)	0.8337 (1)	0.8670 (1)	0.8232 (0)	**0.7040**(− 1)	0.3879 (0)	0.7078
LPSI	0.8181 (1)	**0.9005**(0)	0.7711 (1)	0.7046 (1)	0.5271 (1)	0.3230 (0)	0.0834 (1)	0.5100
NIQE	0.9080 (1)	0.8472 (0)	0.8711 (1)	0.7966 (− 1)	0.8320 (0)	0.6568 (− 1)	0.4498 (0)	0.7450
ILNIQE	0.8972 (1)	0.7384 (1)	0.8794 (1)	0.8422 (1)	0.8553 (1)	0.3057 (0)	0.4389 (0)	0.7185
SNP-NIQE	0.9086 (1)	0.8908 (0)	0.9024 (1)	0.8571 (− 1)	0.8738 (− 1)	0.7155 (− 1)	0.4652 (0)	0.7770
Proposed	**0.9238**	0.8931	**0.9123**	**0.8712**	**0.8997**	**0.7989**	**0.5178**	**0.8087**

**Table 2:** Feature dimension and computational complexity of different unsupervised BIQA methods.

SROCC	Feat. Dim	Time (s)
LPSI	1	0.03
NIQE	36	0.15
ILNIQE	430	2.53
SNP-NIQE	52	2.64
Proposed	71	1.73

### Transform features

Considering that the transform technologies are widely utilized in image processing, we extract transform features from KLT and DCT coefficients to measure image perceptual quality from different aspects.

KLT is a data-driven transform and can extract quality-aware features^12,38^. Non-overlapping patches of MSCN normalized pristine image with size $\sqrt{k}\times \sqrt{k}$ are used to collect vectorized patches to calculate the covariance matrix. The transform kernel $\textbf{P}$ with size $k\times k$ is the eigenvectors of the covariance matrix arranged in descending order based on the eigenvalues. We set *k* to 4 in this paper, and GGD is adopted to fit the KLT coefficient distribution in each frequency band as transform feature.

According to Benford’s law, for a carry system with *b* as the base, the probability of occurrence of a number starting with *n* is $p(n) = log_b{(n+1)}-log_b{(n)}$, and Ou et al.^39^ found that the distance between the distribution of pristine image *p*(*n*) and the distorted image $p_d(n)$ in the DCT domain is highly correlated with the subjective score of the distorted image. Therefore, we calculate the Euclidean distance between these two distributions in the color relevant space in Eq. (5) as features. An example of Benford’s law of distorted image is shown in Fig. 2, we can see that the distribution of DCT coefficient from reference image is very close to the distribution of Benford’s law, however, the distribution of distorted images are quite different.

### Significant feature selection and model training

We extract all the above-mentioned spatial and transform features from two scales, i.e., the original scale, and the 1/2 downsampled scale, except for the DCT feature, which is extracted only from the downsampled scale (for lower computational complexity). The original patch size is 96 $\times$ 96. To reduce the redundancy among different types of features, we select the columns from the feature matrix extracted from 125 pristine natural scene images in^29^ whose average values are higher than 0.01 as significant features. These pristine images are also utilized to train the benchmark MVG model parameters and KLT kernels.

**Table 3:** SROCC results with all distortion types on the full TID2013 database, the best and the second-best results in the unsupervised methods are boldfaced and underlined.

TID2013	AGN	ANC	SCN	MN	FN	IN	QN	GB	DEN	JPEG	JP2K	JGTE	J2TE
BRISQUE	0.674	0.550	0.804	0.222	0.824	0.749	0.677	0.855	0.492	0.751	0.696	0.285	0.719
CORNIA	0.496	0.130	0.655	0.373	0.715	0.647	0.632	0.844	0.688	0.758	0.866	0.587	0.603
MEON	0.813	0.722	0.926	0.728	0.911	0.901	0.888	0.887	0.797	0.850	0.891	0.746	0.716
RankIQA	0.667	0.620	0.821	0.365	0.760	0.736	0.783	0.809	0.767	0.866	0.878	0.704	0.810
LPSI	0.769	0.495	0.697	0.046	**0.925**	0.432	0.854	0.841	0.249	**0.912**	0.899	0.091	**0.611**
NIQE	0.815	0.591	0.541	0.721	0.851	0.745	0.861	0.810	0.578	0.860	0.866	0.122	0.400
ILNIQE	0.876	**0.814**	**0.923**	0.512	0.869	0.753	**0.873**	0.814	**0.748**	0.835	0.861	0.274	0.523
SNP-NIQE	**0.886**	0.733	0.650	**0.740**	0.873	**0.800**	0.857	**0.864**	0.613	0.879	0.878	0.282	0.592
Proposed	0.822	0.697	0.775	0.694	0.849	0.761	0.856	0.831	0.702	0.889	**0.910**	**0.342**	0.421

TID2013	NEPN	BLOCK	MS	CC	CCS	MGN	CN	LCNI	ICQD	CHA	SSR	Avg.	
BRISQUE	0.158	0.362	0.253	0.102	0.200	0.587	0.211	0.546	0.842	0.770	0.764	0.546	
CORNIA	0.282	0.025	0.194	0.145	0.006	0.461	0.560	0.648	0.646	0.672	0.867	0.520	
MEON	0.116	0.500	0.177	0.252	0.684	0.849	0.406	0.772	0.857	0.779	0.855	0.709	
RankIQA	0.512	0.622	0.268	0.613	0.662	0.619	0.644	0.800	0.779	0.629	0.859	0.691	
LPSI	0.052	0.137	**0.341**	**0.199**	**0.302**	0.696	0.018	0.236	**0.900**	**0.695**	0.862	0.511	
NIQE	0.033	**0.174**	0.147	0.106	**0.302**	0.679	0.054	0.754	0.760	0.568	0.852	0.550	
ILNIQE	**0.080**	0.130	0.182	0.014	0.166	0.690	**0.354**	0.829	0.749	0.680	0.865	**0.601**	
SNP-NIQE	0.015	0.032	0.100	0.156	0.106	**0.740**	0.208	**0.830**	0.790	0.635	0.829	0.587	
Proposed	0.017	0.146	0.024	0.185	0.104	0.655	0.291	0.808	0.844	0.641	**0.888**	0.590

**Table 4:** SROCC results with all distortion types on the full KADID-10k database, the best and the second-best results in the unsupervised methods are boldfaced and underlined.

KADID-10k	GB	LB	MB	CD	CS	CQ	CSA1	CSA2	JP2K	JPEG	WN	WNCC	IN
BRISQUE	0.879	0.840	0.780	0.527	0.031	0.777	0.012	0.349	0.663	0.803	0.653	0.644	0.824
CORNIA	0.886	0.885	0.805	0.511	0.213	0.818	0.203	0.567	0.678	0.812	0.774	0.846	0.776
LPSI	0.892	0.788	0.429	0.353	0.000	0.622	**0.106**	0.095	0.828	0.846	0.512	0.611	0.113
NIQE	0.879	0.843	0.755	0.320	**0.291**	0.643	0.011	0.059	0.807	0.813	0.704	0.786	0.804
ILNIQE	0.883	0.846	**0.779**	**0.671**	0.090	0.676	0.027	**0.677**	0.790	0.804	0.776	0.841	0.808
SNP-NIQE	**0.908**	**0.860**	0.740	0.297	0.279	0.689	0.076	0.091	0.823	0.837	**0.835**	**0.886**	0.819
Proposed	0.904	0.839	0.763	0.275	0.163	**0.711**	0.104	0.354	**0.876**	**0.856**	0.729	0.795	**0.830**

KADID-10k	MN	DEN	Brighten	Darken	MS	Jitter	NEP	PIX	QN	CB	HS	CC	Avg.
BRISQUE	0.601	0.855	0.467	0.283	0.046	0.815	0.274	0.825	0.473	0.154	0.693	0.111	0.535
CORNIA	0.836	0.662	0.684	0.524	0.238	0.797	0.148	0.621	0.483	0.305	0.774	0.172	0.601
LPSI	0.552	0.100	0.385	0.158	0.055	0.723	0.112	**0.747**	0.642	**0.034**	0.513	0.016	0.409
NIQE	0.681	0.595	0.323	0.287	0.029	0.768	**0.275**	0.714	**0.752**	0.007	0.572	0.013	0.509
ILNIQE	0.682	**0.856**	0.301	0.436	**0.315**	0.441	0.218	0.577	0.571	0.003	0.681	0.072	**0.553**
SNP-NIQE	**0.688**	0.651	0.303	0.259	0.005	0.792	0.236	0.698	0.722	0.025	**0.718**	0.020	0.530
Proposed	0.675	0.353	**0.472**	**0.468**	0.179	**0.843**	0.270	0.725	0.483	0.018	0.714	**0.092**	0.540

**Figure 3:**
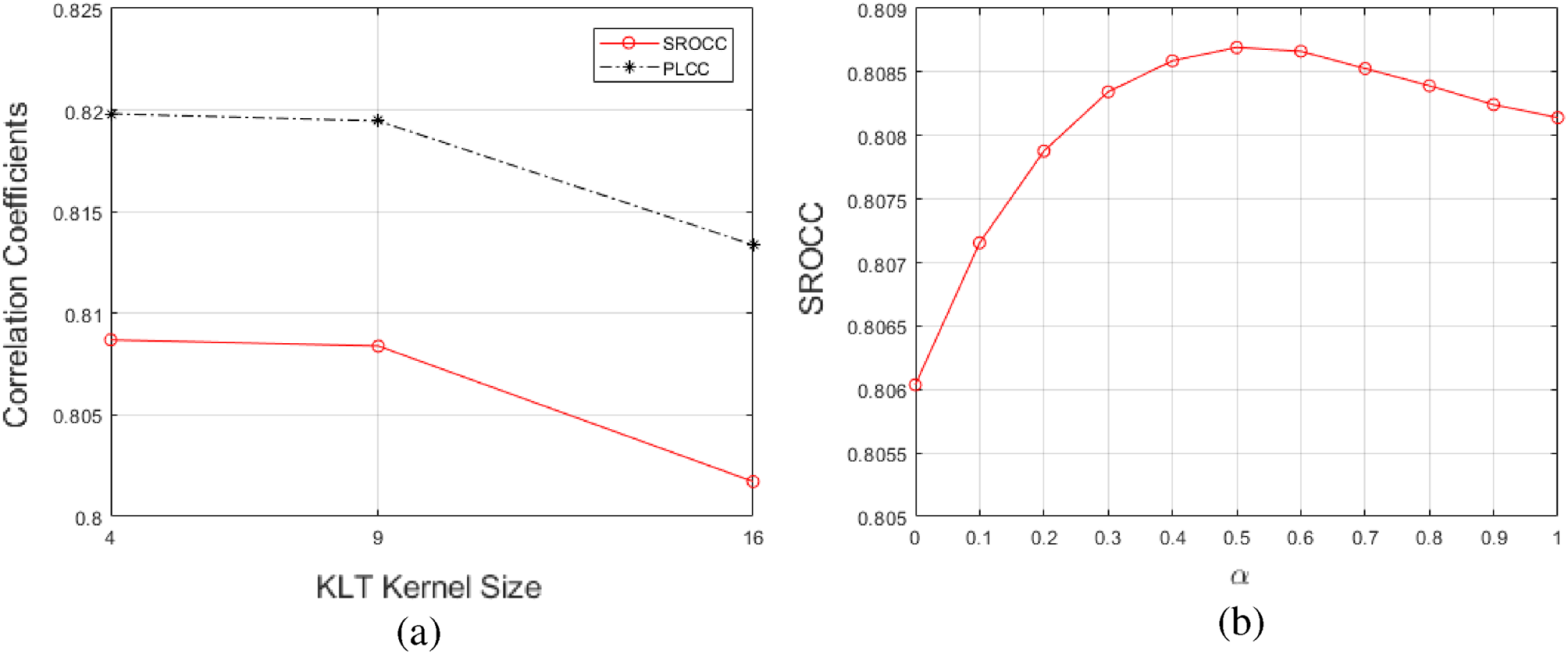
Parameters optimization: (**a**) performance with different KLT kernel sizes, (**b**) performance with different global/local fusion weights.

**Table 5:** Feature selection of GM-LOG on seven IQA databases, the best results are boldfaced.

$TH_{GM-LOG}$	–	0	0.01	0.05	–
GM-Log Dim.	80	16	12	6	0
LIVE					
SROCC	**0.9290**	0.9286	0.9266	0.9238	0.9050
KROCC	**0.7635**	0.7634	0.7609	0.7566	0.7283
PLCC	**0.9278**	0.9259	0.9238	0.9209	0.9049
RMSE	**10.1961**	10.3230	10.4596	10.6530	11.6313
MICT					
SROCC	0.8799	**0.8972**	0.8933	0.8931	0.8824
KROCC	0.6965	**0.7178**	0.7118	0.7122	0.6962
PLCC	0.8800	**0.8973**	0.8929	0.8900	0.8709
RMSE	0.4875	**0.4530**	0.4621	0.4681	0.5044
CSIQ					
SROCC	0.9025	0.9091	0.9113	**0.9123**	0.9009
KROCC	0.7278	0.7357	0.7379	**0.7389**	0.7175
PLCC	0.8749	0.8773	0.8777	**0.8825**	0.8438
RMSE	0.1369	0.1356	0.1354	**0.1329**	0.1517
TID2013					
SROCC	0.8462	0.8612	0.8690	0.8712	**0.8763**
KROCC	0.6607	0.6793	0.6878	**0.6895**	0.6893
PLCC	0.8733	0.8886	**0.8951**	0.8836	0.8868
RMSE	0.6795	0.6397	**0.6219**	0.6590	0.6447
Kadid-10k					
SROCC	0.8629	0.8876	0.8922	**0.8997**	0.8947
KROCC	0.6740	0.7026	0.7080	**0.7174**	0.7052
PLCC	0.8913	0.9077	0.8276	**0.9197**	0.9125
RMSE	0.5335	0.4936	0.6823	**0.4620**	0.4812
CID2013					
SROCC	0.7846	0.7914	0.7945	**0.7989**	0.7949
KROCC	0.5861	0.5927	0.5950	**0.5991**	0.5954
PLCC	0.7945	0.8006	0.8030	**0.8067**	0.1162
RMSE	13.7482	13.5668	13.4947	**13.3782**	22.4870
LIVE-C					
SROCC	0.5062	0.5147	0.5176	**0.5178**	0.5159
KROCC	0.3482	0.3524	**0.3545**	0.3544	0.3530
PLCC	0.5303	0.5422	0.5458	**0.5481**	0.5473
RMSE	17.1918	17.0387	16.9921	**16.9608**	16.9720

## Experimental results

The experiments are conducted on eight widely utilized IQA databases, including natural scene IQA databases LIVE^40^, MICT^41^, CSIQ^42^, TID2013^43^, KADID-10k^44^, CID2013^45^, LIVE Challenge (LIVE-C)^46^, as well as screen content IQA databases SIQAD^47^. All of these databases are publicly available.

### Comparison with state-of-the-art methods

Table 1 shows the Spearman Rank Order Correlation Coefficient (SROCC) of the proposed method as well as supervised BIQA methods^8,16,48,49^ and unsupervised methods^29–31^ on common distortion types (JPEG compression, JPEG2000 compression, white noise, and Gaussian blur) and real-world distortion. Specifically, JPEG and JPEG2000 compression refer to lossy image compression using the JPEG and JPEG2000 codecs, which are very common in image compression. White noise refers to additive white Gaussian noise, which is commonly encountered in image acquisition and transmission. Gaussian blur refers to blurring an image using a Gaussian filter, which is quite common in image acquisition. LIVE, MICT, CSIQ_sub, TID2013_sub, and KADID-10k_sub have common distortions. CID2013 and LIVE-C have real-world distortion. Specifically, the SROCC values in each row are calculated between the objective scores predicted by the method and the subjective scores that are given in the databases. We utilize LIVE to train the supervised models and test on the remaining six IQA databases. “W. A.” refers to the weighted average results on the above seven IQA databases with the number of distorted images in each database being the weights. The best results of both supervised and unsupervised methods are boldfaced. We can see that our method achieves the highest results on both common and authentic distortions, as well as the weighted average result compared to reference methods.

We also perform statistical analysis by applying t-test on the prediction residuals, the results are also tabulated in Table 1, wherein (1), (0), and (− 1) tell that our method is superior, comparative, and inferior to the reference method statistically with 95% confidence. According to^31^, the residuals are obtained by calculating the differences between the subjective scores and the converted objective scores using Eq. (11):

11 $$\begin{aligned} s(q)=\beta _1\left( \frac{1}{2}-\frac{1}{1+e^{\beta _2(q-\beta _3)}}\right) +\beta _4 q + \beta _5, \end{aligned}$$

where *q* and *s*(*q*) are the objective and the converted scores, respectively, $\beta _1$–$\beta _5$ are the curve fitting parameters. This conversion is necessary because objective and subjective scores have different scales. The proposed method achieves comparable results with NIQE and SNP-NIQE, and better results than ILNIQE on common distortions.

Table 2 shows the feature dimension and average extraction time of different unsupervised BIQA methods. The test methods are all implemented with MATLAB and tested on Windows system with Intel Core i7-3770 3.40 GHz dual-core CPU and 8 GB RAM. ILNIQE has good generalization performance but its computational complexity is too high. LPSI has very low computational complexity, but its performance is limited. The proposed method achieves the highest average results on seven databases, and the generalization performance and running time are comparative.

The SROCC results of all distortions on TID2013 and KADID-10k are tabulated in Tables 3 and 4, respectively. For better visualization, the best and the second-best results in the unsupervised methods are boldfaced and underlined. The proposed method achieved comparable average performance with state-of-the-art unsupervised BIQA methods on both databases, which has demonstrated the good performance of our method on uncommon distortion. However, the proposed method has failure cases on some specific distortions, such as non-eccentricity pattern noise (NERP) and mean shift (MS) in Table 3, which are luminance-related distortions, and change of color saturation (CCS) in Table 3, color diffusion (CD), denoise (DEN), and quantization (QN) in Table 4, which are color-related distortions. Since the features utilized in our method mainly measure image structure and naturalness, we cannot accurately quantify luminance and color-related distortions, which is a shortcoming of our method and can be addressed in future work.

From these tables, we can see that with the joint utilization of finely selected spatial and transform features, the proposed method can assess the image perceived quality from different aspects, resulting in achieving the highest quality assessment accuracy on most distortion types with relatively low computational complexity. The results have shown the superiority and good generalization capability of our method as an unsupervised BIQA method.

### Parameters optimization and ablation studies

The original GM-LOG features have many zeros and outliers which make it less efficient, therefore we attempt to remove these redundant features to improve the efficiency of GM-LOG. We perform the GM-LOG feature selection based on selecting the features with their minimum values higher than $TH_{GM-LOG}$, the indices of selected features are obtained from pristine images^29^, and then applied to features extracted from distorted images. The results of GM-LOG features selected with different $TH_{sf}$ values are tabulated in Table 5, with the best results boldfaced. We can see from the table that when setting $TH_{sf}$ = 0.05, the proposed method achieves the highest results on CSIQ, Kadid-10k, CID2013, and LIVE-C, and comparable results on the rest three databases. Therefore, in consideration of both efficiency and feature dimension, we set $TH_{sf}$ = 0.05 to select efficient GM-LOG features in this paper.

We conduct experiments on seven IQA databases to study the optimal KLT kernel size for transform feature extraction, and employ SROCC as well as Kendall rank-order correlation coefficient (KROCC) and Pearson linear correlation coefficient (PLCC) to evaluate the performance. The objective scores are mapped to the subjective scores via nonlinear mapping in Eq. (11) before calculating PLCC^50^. Weighted average results on seven IQA databases are shown in Fig. 3a, we can see that with the increase of KLT kernel size (from 4 $\times$ 4 to 16 $\times$ 16), the three metrics decrease, therefore we set *k* = 4 in this paper. We also conduct experiments on seven IQA databases to study the impact of $\alpha$ in Eq. (3) and plot the weighted average SROCC results on Fig. 3b, we can see that when setting $\alpha$ = 0.5, the proposed achieved the highest result on seven databases.

**Table 6:** Performances on different IQA databases with different significant features selection thresholds, the best results are boldfaced.

$TH_{sf}$		0.05	0.01	0
Feat. Dim		67	71	77
LIVE	SROCC	0.9236	0.9238	**0.9248**
	KROCC	0.7560	0.7566	**0.7576**
	PLCC	0.9209	0.9209	**0.9219**
	RMSE	10.6503	10.6530	**10.5879**
MICT	SROCC	0.8909	**0.8931**	0.8921
	KROCC	0.7081	**0.7122**	0.7114
	PLCC	0.8835	**0.8900**	0.8855
	RMSE	0.4808	**0.4681**	0.4769
CSIQ	SROCC	**0.9134**	0.9123	0.9128
	KROCC	**0.7416**	0.7389	0.7396
	PLCC	**0.8845**	0.8825	0.8792
	RMSE	**0.1318**	0.1329	0.1346
TID2013	SROCC	0.8658	**0.8712**	0.8685
	KROCC	0.6838	**0.6895**	0.6866
	PLCC	0.8922	**0.8836**	0.8911
	RMSE	0.6300	**0.6590**	0.6331
Kadid-10k	SROCC	0.8938	0.8997	**0.8953**
	KROCC	0.7115	0.7174	**0.7113**
	PLCC	0.9165	0.9197	**0.8275**
	RMSE	0.4708	0.4620	**0.6605**
CID2013	SROCC	**0.7998**	0.7989	0.7946
	KROCC	**0.6010**	0.5991	0.5948
	PLCC	**0.8077**	0.8067	0.8036
	RMSE	**13.3476**	13.378	13.4736
LIVE-C	SROCC	0.5157	**0.5178**	0.5145
	KROCC	0.3522	**0.3544**	0.3528
	PLCC	0.5469	0.5481	**0.5493**
	RMSE	16.9768	16.9608	**16.9454**

**Table 7:** SROCC results of each type of feature in the proposed method, the best results are boldfaced.

Feat. Dim.	52	19	71
Database	Spatial	Transform	Combined
LIVE	**0.9348**	0.8422	0.9238
MICT	0.8846	0.6552	**0.8931**
CSIQ	0.8946	0.8529	**0.9123**
TID2013	0.8447	0.7950	**0.8712**
TID2013 (full)	0.3822	**0.4287**	0.4001
KADID-10k	0.8782	0.8175	**0.8997**
KADID-10k (full)	0.3483	**0.4019**	0.3861
CID2013	0.7925	0.7091	**0.7989**
LIVE-C	0.4991	0.4553	**0.5178**

As we extracted multiple features as spatial and transform features, some features may contribute less than the other features. therefore, to further improve the performance and reduce the feature dimension, we perform significant feature selection to remove the insignificant features. The experiment is conducted on seven IQA databases, and we remove the insignificant features by selecting the features with their average values higher than $TH_{sf}$. We mark these feature indices based on features extracted from pristine images^29^, and then test on IQA databases, the results are tabulated in Table 6, with the best results boldfaced. We can see from the table that when setting $TH_{sf}$ = 0.01, the proposed method achieves the highest results on MICT, TID2013, and LIVE-C, and comparable results on the rest four databases.

To verify the effectiveness of the two types of features, i.e. spatial features and transform features, we report the ablation test results in Table 7, where we can see that the spatial features usually take the leading role and transform features work as supplements, and the combination of these two features achieves the optimal results. However, we can also see that the performance of combined features is inferior to the single transform feature, but better than the single spatial feature on full TID2013 and KADID-10k databases, which means the transform feature takes the leading role on uncommon artificial distortion types.

### Performance on screen content images

**Table 8:** Performance comparison of unsupervised BIQA methods on SIQAD, the best results are boldfaced.

	SROCC	KROCC	PLCC	RMSE
LPSI	0.2914	0.2081	0.3516	13.3999
PIQE	0.3297	0.2264	0.4605	12.7062
NIQE	0.3585	0.2472	0.3800	13.2405
ILNIQE	0.3197	0.2263	0.3885	13.1897
SNP-NIQE	0.4647	0.3221	0.4748	12.5974
Proposed	**0.5288**	**0.3791**	**0.5665**	**11.7959**

The proposed method is designed for natural scene images, we also conduct an experiment on the screen image quality assessment database (SIQAD)^47^ to further verify the generalization ability. SROCC, KROCC, PLCC, and root mean square error (RMSE) of different unsupervised BIQA methods are tabulated in Table 8, with the best results boldfaced. Similarly, the objective scores are mapped to the subjective scores via nonlinear mapping in Eq. (11) before calculating RMSE^50^. We can see from the table that the proposed method achieved the best performance compared with competing unsupervised BIQA methods, but the result is still not good enough and can be further improved in the future.

## Conclusion

In this paper, we proposed an unsupervised BIQA method utilizing joint spatial and transform features. Specifically, we utilized PC, GM, GM-LOG and MSCN as spatial features, and KLT and DCT coefficients as transform features. And these features were well analyzed to remove the redundancy and then fitted to MVG model for unsupervised BIQA. Experiments on multiple IQA databases indicated that the proposed method achieved state-of-the-art results with low complexity on both artificial and authentic distortions. Future work will be focused on improving the performance on authentic distortions and illustrated images.

## Data Availability

The datasets used and/or analyzed during the current study are available from the corresponding author upon reasonable request. And we clarify that no human or animal is directly involved during this study.

## References

[1] Wang Z, Bovik A, Sheikh H, Simoncelli E (2004). Image quality assessment: From error visibility to structural similarity. IEEE Trans. Image Process..

[2] Zhang L, Zhang L, Mou X, Zhang D (2011). FSIM: A feature similarity index for image quality assessment. IEEE Trans. Image Process..

[3] Jain, A. & Bhateja, V. A full-reference image quality metric for objective evaluation in spatial domain. In *2011 International Conference on Communication and Industrial Application*, 1–5 (2011).

[4] Kim, J. & Lee, S. Deep blind image quality assessment by employing FR-IQA. In *2017 IEEE International Conference on Image Processing (ICIP)*, 3180–3184 (2017).

[5] Wu, J., Liu, Y., Shi, G. & Lin, W. Saliency change based reduced reference image quality assessment. In *2017 IEEE Visual Communications and Image Processing (VCIP)*, 1–4 (2017).

[6] Liu Y (2018). Reduced-reference image quality assessment in free-energy principle and sparse representation. IEEE Trans. Multimed..

[7] Hu, Q., Sheng, Y., Yang, L., Li, Q. & Chai, L. Reduced-reference image quality assessment for single-image super-resolution based on wavelet domain. In *2019 Chinese Control And Decision Conference (CCDC)*, 2067–2071 (2019).

[8] Mittal A, Moorthy AK, Bovik AC (2012). No-reference image quality assessment in the spatial domain. IEEE Trans. Image Process..

[9] Wu, Q., Wang, Z. & Li, H. A highly efficient method for blind image quality assessment. In *2015 IEEE International Conference on Image Processing (ICIP)*, 339–343 (2015).

[10] Ma H, Cui Z, Gan Z, Tang G, Liu F (2022). Saliency-enhanced two-stream convolutional network for no-reference image quality assessment. J. Electron. Imaging.

[11] Sang Q (2021). MoNET: No-reference image quality assessment based on a multi-depth output network. J. Electron. Imaging.

[12] Yang C, Zhang X, An P, Shen L, Kuo C-CJ (2021). Blind image quality assessment based on multi-scale KLT. IEEE Trans. Multimed..

[13] Yang C, An P, Shen L (2022). Blind image quality measurement via data-driven transform-based feature enhancement. IEEE Trans. Instrum. Meas..

[14] Zhang M, Muramatsu C, Zhou X, Hara T, Fujita H (2015). Blind image quality assessment using the joint statistics of generalized local binary pattern. IEEE Signal Process. Lett..

[15] Min X, Zhai G, Gu K, Liu Y, Yang X (2018). Blind image quality estimation via distortion aggravation. IEEE Trans. Broadcast..

[16] Ma K (2018). End-to-end blind image quality assessment using deep neural networks. IEEE Trans. Image Process..

[17] Zhu, H., Li, L., Wu, J., Dong, W. & Shi, G. MetaIQA: Deep meta-learning for no-reference image quality assessment. In *2020 IEEE/CVF Conference on Computer Vision and Pattern Recognition (CVPR)*, 14131–14140 (2020).

[18] Wang, X., Xiong, J., Li, B., Suo, J. & Gao, H. Learning hybrid representations of semantics and distortion for blind image quality assessment. In *ICASSP 2023– 2023 IEEE International Conference on Acoustics, Speech and Signal Processing (ICASSP)*, 1–5 (2023).

[19] Lan, X. *et al.* Multilevel feature fusion for end-to-end blind image quality assessment. *IEEE Transactions on Broadcasting* 1–11 (2023).

[20] Wang H, Tu Y, Liu X, Tan H, Liu H (2023). Deep ordinal regression framework for no-reference image quality assessment. IEEE Signal Process. Lett..

[21] Madhusudana PC, Birkbeck N, Wang Y, Adsumilli B, Bovik AC (2022). Image quality assessment using contrastive learning. IEEE Trans. Image Process..

[22] Pan Z (2022). DACNN: Blind image quality assessment via a distortion-aware convolutional neural network. IEEE Trans. Circ. Syst. Video Technol..

[23] Chen B (2022). No-reference image quality assessment by hallucinating pristine features. IEEE Trans. Image Process..

[24] Pan Z (2023). No-reference image quality assessment via multibranch convolutional neural networks. IEEE Trans. Arti. Intell..

[25] Zhou M (2023). An end-to-end blind image quality assessment method using a recurrent network and self-attention. IEEE Trans. Broadcast..

[26] Liu M, Huang J, Zeng D, Ding X, Paisley J (2023). A multiscale approach to deep blind image quality assessment. IEEE Trans. Image Process..

[27] Cao Y, Min X, Sun W, Zhai G (2023). Attention-guided neural networks for full-reference and no-reference audio-visual quality assessment. IEEE Trans. Image Process..

[28] Yu L, Li J, Pakdaman F, Ling M, Gabbouj M (2023). MAMIQA: No-reference image quality assessment based on multiscale attention mechanism with natural scene statistics. IEEE Signal Process. Lett..

[29] Mittal A, Soundararajan R, Bovik AC (2013). Making a “completely blind” image quality analyzer. IEEE Signal Process. Lett..

[30] Zhang L, Zhang L, Bovik AC (2015). A feature-enriched completely blind image quality evaluator. IEEE Trans. Image Process..

[31] Liu Y (2020). Unsupervised blind image quality evaluation via statistical measurements of structure, naturalness, and perception. IEEE Trans. Circ. Syst. Video Technol..

[32] N, V., D, P., Bh, M. C., Channappayya, S. S. & Medasani, S. S. Blind image quality evaluation using perception based features. In *2015 Twenty First National Conference on Communications (NCC)*, 1–6 (2015).

[33] Wu L, Zhang X, Chen H, Zhou Y (2020). Unsupervised quaternion model for blind colour image quality assessment. Signal Process..

[34] Wu L, Zhang X, Chen H, Wang D, Deng J (2021). VP-NIQE: An opinion-unaware visual perception natural image quality evaluator. Neurocomputing.

[35] Kovesi P (1999). Image features from phase congruency. Videre J. Comput. Vis. Res..

[36] Geusebroek J, van den Boomgaard R, Smeulders AWM, Geerts H (2001). Color invariance. IEEE Trans. Pattern Anal. Mach. Intell..

[37] Sharifi K, Leon-Garcia A (1995). Estimation of shape parameter for generalized Gaussian distributions in subband decompositions of video. IEEE Trans. Circ. Syst. Video Technol..

[38] Zhang X, Kwong S, Kuo C-CJ (2021). Data-driven transform-based compressed image quality assessment. IEEE Trans. Circ. Syst. Video Technol..

[39] Ou, F.-Z., Wang, Y.-G. & Zhu, G. A novel blind image quality assessment method based on refined natural scene statistics. In *2019 IEEE International Conference on Image Processing (ICIP)*, 1004–1008 (2019).

[40] Sheikh, H. R., Wang, Z., Cormack, L. & Bovik, A. C. LIVE image quality assessment database release 2 (2005). http://live.ece.utexas.edu/research/quality.

[41] Horita, Y., Shibata, K. & Kawayoka, Y. Toyama Image quality evaluation database (2011). http://mict.eng.u-toyama.ac.jp/mictdb.html.

[42] Larson, E. & Chandler, D. Categorical image quality (CSIQ) database (2010). http://vision.okstate.edu/csiq.

[43] Ponomarenko, N. *et al.* Color image database TID2013: Peculiarities and preliminary results. In *European Workshop on Visual Information Processing (EUVIP)*, 106–111 (2013). http://www.ponomarenko.info/tid2013.htm.

[44] Lin, H., Hosu, V. & Saupe, D. KADID-10k: A large-scale artificially distorted IQA database. In *2019 Eleventh International Conference on Quality of Multimedia Experience (QoMEX)*, 1–3 (2019). http://database.mmsp-kn.de/kadid-10k-database.html.

[45] Virtanen T, Nuutinen M, Vaahteranoksa M, Oittinen P, Häkkinen J (2015). CID2013: A database for evaluating no-reference image quality assessment algorithms. IEEE Trans. Image Process..

[46] Ghadiyaram D, Bovik AC (2016). Massive online crowdsourced study of subjective and objective picture quality. IEEE Trans. Image Process..

[47] Yang H, Fang Y, Lin W (2015). Perceptual quality assessment of screen content images. IEEE Trans. Image Process..

[48] Ye, P., Kumar, J., Kang, L. & Doermann, D. Unsupervised feature learning framework for no-reference image quality assessment. In *2012 IEEE Conference on Computer Vision and Pattern Recognition*, 1098–1105 (2012).

[49] Liu, X., Van De Weijer, J. & Bagdanov, A. D. RankIQA: Learning from rankings for no-reference image quality assessment. In *2017 IEEE International Conference on Computer Vision (ICCV)*, 1040–1049 (2017).

[50] Antkowiak, J. & Baina, T. J. Final report from the video quality experts group on the validation of objective models of video quality assessment march. *ITU-T Standards Contribution COM* (2000).

